# Arbuscular Mycorrhizal Fungi Improve the Performance of Tempranillo and Cabernet Sauvignon Facing Water Deficit under Current and Future Climatic Conditions

**DOI:** 10.3390/plants13081155

**Published:** 2024-04-22

**Authors:** Daria Kozikova, Inmaculada Pascual, Nieves Goicoechea

**Affiliations:** Plant Stress Physiology Group, Associated Unit to CSIC (EEAD, Zaragoza, Spain), BIOMA Institute for Biodiversity and the Environment, University of Navarra, Irunlarrea, 1, 31008 Pamplona, Spain; dkozikova@alumni.unav.es (D.K.); niegoi@unav.es (N.G.)

**Keywords:** climate change, gas exchange, grapevine, mycorrhizal fungi, water relations

## Abstract

Climate change (CC) threatens Mediterranean viticulture. Rhizospheric microorganisms may be crucial for the adaptation of plants to CC. Our objective was to assess whether the association of two grapevine varieties with arbuscular mycorrhizal fungi (AMF) increases grapevine’s resilience to environmental conditions that combine elevated atmospheric CO_2_, increased air temperatures, and water deficit. Tempranillo (T) and Cabernet Sauvignon (CS) plants, grafted onto R110 rootstocks, either inoculated (+M) or not (−M) with AMF, were grown in temperature-gradient greenhouses under two environmental conditions: (i) current conditions (ca. 400 ppm air CO_2_ concentration plus ambient air temperature, CATA) and (ii) climate change conditions predicted by the year 2100 (700 ppm of CO_2_ plus ambient air temperature +4 °C, CETE). From veraison to maturity, for plants of each variety, inoculation treatment and environmental conditions were also subjected to two levels of water availability: full irrigation (WW) or drought cycles (D). Therefore, the number of treatments applied to each grapevine variety was eight, resulting from the combination of two inoculation treatments (+M and −M), two environmental conditions (CATA and CETE), and two water availabilities (WW and D). In both grapevine varieties, early drought decreased leaf conductance and transpiration under both CATA and CETE conditions and more markedly in +M plants. Photosynthesis did not decrease very much, so the instantaneous water use efficiency (*WUE*) increased, especially in drought +M plants under CETE conditions. The increase in *WUE* coincided with a lower intercellular-to-atmospheric CO_2_ concentration ratio and reduced plant hydraulic conductance. In the long term, mycorrhization induced changes in the stomatal anatomy under water deficit and CETE conditions: density increased in T and decreased in CS, with smaller stomata in the latter. Although some responses were genotype-dependent, the interaction of the rootstock with AMF appeared to be a key factor in the acclimation of the grapevine to water deficit under both current and future CO_2_ and temperature conditions.

## 1. Introduction

According to the predictions of the IPCC [1], Europe, and in particular, the Mediterranean area, is set to be severely affected by climate change in the coming decades. Rainfall is expected to decrease by between 4 and 22%, depending on greenhouse gas emissions, and droughts could become more common in most Mediterranean areas. This significant reduction in water availability is associated with increased air temperatures and more frequent and intense heat waves, making it urgent to find alternatives and solutions for viticulture, which is seriously threatened in some of the world’s leading wine-producing countries, namely Italy, France, and Spain (OIV 2019) [2]. Phenology, vegetative development, physiological and biochemical performance, as well as cluster quality and quantity are aspects highly sensitive to weather extremes such as drought and high temperatures [3,4,5]. 

Tempranillo is the most important red grape variety in Spain and is used to produce some of the most prestigious wines from Spanish appellations, such as Rioja, Ribera del Duero, and Toro. In 2021, it was grown on approximately 202,917 hectares, representing 21% of the total vineyard area [6]. This early-ripening red variety is also grown in other countries around the world, such as Portugal, the USA, France, Australia, and Argentina, where it is known by other names such as Aragonez, Valdepeñas or Tinta Roriz [7]. Cabernet Sauvignon, a late-maturing variety that almost certainly originated in Bordeaux, France, is one of the most prestigious red grapes in the world [5]. In fact, there are Cabernet plantations in the world’s major wine-producing countries: France, Spain, Italy, the USA, Chile, Argentina, and New Zealand.

Simonneau et al. [8] considered rootstocks as the “hidden half” part of the plant material, which can offer more flexible solutions than the selection of grapevine varieties for adapting vineyards to water deficit. Indeed, several studies [9,10] have evaluated the performance of different grapevine rootstocks under drought conditions. Among the rootstocks tested, 110R has been classified as drought tolerant [11] due to its adequate hydraulic properties to withstand the water deficit, which is a key factor that can determine the degree of drought or waterlogging resistance to grapevine rootstocks [12]. Three of the main aspects involved in the hydraulic properties of rootstocks are (i) root development, (ii) the control of water transport from roots to shoots, and (iii) the presence of aquaporins for inter- and intracellular water transport [8]. The symbiotic association of roots with arbuscular mycorrhizal fungi (AMF) can modulate these three aspects since mycorrhized roots can (i) explore a larger volume of soil, improving water and mineral uptake, as well as mycorrhizal symbiosis [13]; (ii) increase soil–plant hydraulic conductance [14]; and (iii) downregulate the expression of genes encoding aquaporins, thus anticipating their downregulation in plants exposed to drought [15]. Furthermore, the cooperation between AMF and plant-growth-promoting rhizobacteria (PGPR) can have synergistic effects on root morphology [16]. In a field study conducted in Italy at the beginning of summer, when environmental temperatures were rising and water availability was decreasing, planted grapevine rootstocks showed higher survival rates and improved growth when associated with AMF [17]. Mycorrhization also increased the presence of beneficial taxa and influenced soil bacterial communities. According to Darriaut et al. [18], the interactions between the vine rootstock and the soil microbiome (mainly fungi and bacteria) may be crucial for resilient viticulture in the face of climate change.

Considering all these aspects, the objectives of our work were (i) to evaluate to what extent the application of AMF in a mixture with PGPR can improve the physiological performance of Tempranillo and Cabernet Sauvignon grafted on the drought-resistant rootstock 110R facing limited water availability under current or future air temperature and CO_2_ concentration, and (ii) to assess whether the variety of grapevine grafted on the 110R rootstock can influence the performance of the whole plant.

## 2. Results

### 2.1. Percentage of Mycorrhizal Colonization

The percentage of AMF colonization was determined in the roots before imposing different environmental conditions. The percentage of AMF colonization was high and similar in both varieties, reaching values of 75.9% in T and 76.2% in CS.

### 2.2. Physiological and Anatomical Responses to Water Deficit under Different Environmental Conditions

**Figure 1:**
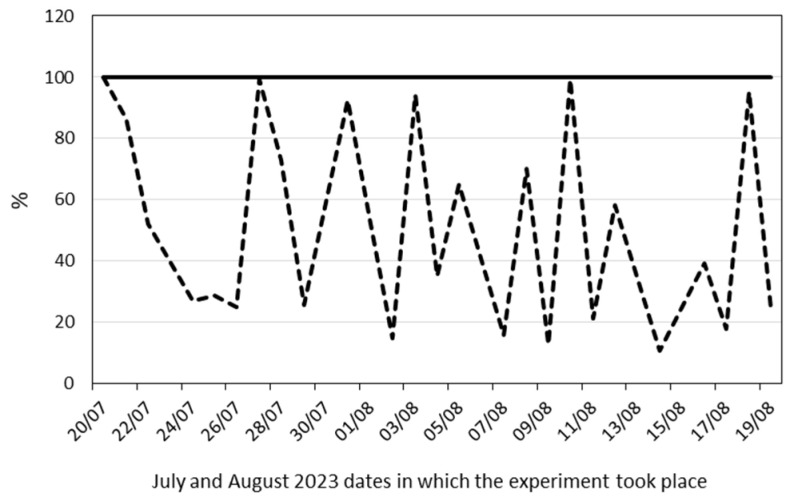
Evolution of the substrate water content from fruit veraison to maturity. Dashed line, percentage of water in droughted pots; solid line, well-watered control pots (100%).

Figure 1 shows the evolution of the water content in the substrate during the drought cycles imposed from veraison to grape maturity. The dashed line represents the percentage of water in droughted pots compared to well-watered (WW) controls (solid line, 100%). 

The results obtained in this study indicate that the plant responses to water deficit under the current and predicted atmospheric conditions (CO_2_ and temperature) could be classified as ‘early’ and ‘late’ responses. Early responses refer to those occurring during the first two weeks after the beginning of the drought cycles and late responses to those observed during fruit ripening (approximately 2.5 months later). Moreover, since all the plants received a mixture of PGPR, the differences between −M and +M plants could be attributed to the absence (−M) or the presence (+M) of AMF. 

#### 2.2.1. Early Responses

**Figure 2:**
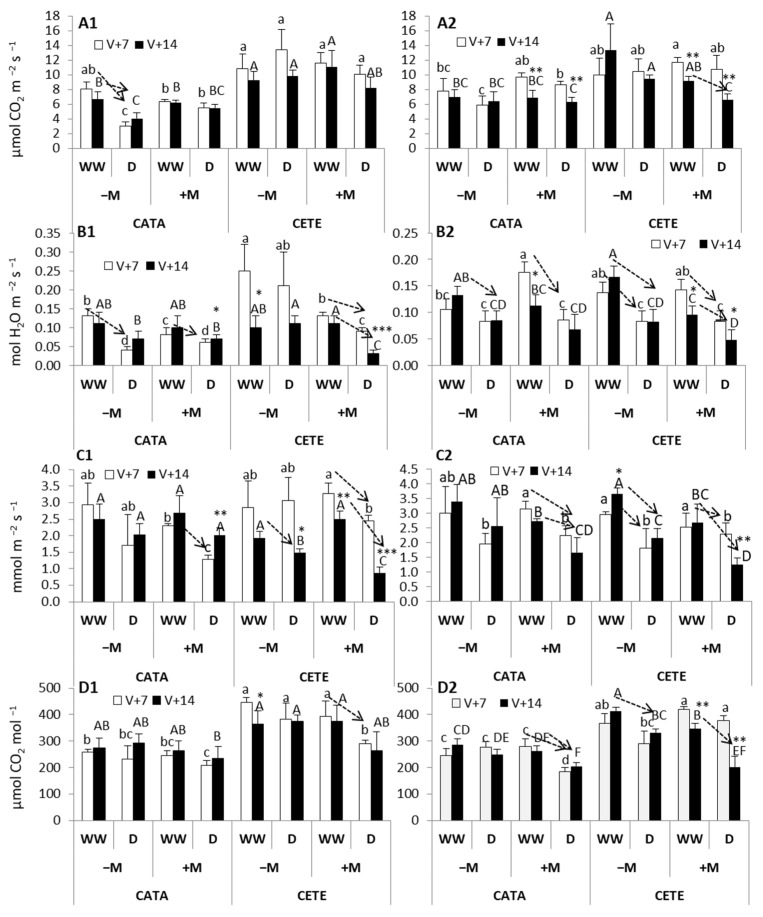
(**A**) Photosynthesis, (**B**) leaf conductance, (**C**) transpiration, and (**D**) intercellular CO_2_ concentration on days 7 (white bars) and 14 (black bars) after the onset of drought in Tempranillo (1) and Cabernet Sauvignon (2). Bars represent means (*n* = 4) ± SD. For each gas exchange parameter and grapevine variety, different lowercase and capital letters indicate significant differences (*p* ≤ 0.05) on days 7 and 14, respectively, among the different combinations of treatments (non-mycorrhizal, −M; mycorrhizal, +M; well-watered, WW; drought, D; current CO_2_ and temperature, CATA; and predicted CETE). Asterisks indicate significant differences (*, *p* ≤ 0.05; **, *p* ≤ 0.01; ***, *p* ≤ 0.001) between days 7 and 14. For each variety (T or CS), environmental condition (CATA or CETE), and mycorrhizal inoculation (−M or +M) arrows highlight significant differences between WW and D treatments.

Figure 2 shows the values of photosynthesis (An), leaf conductance (gs), transpiration (E), and intercellular CO_2_ concentration (Ci) in the different varieties and treatments. In order to highlight the significant effects of water deficit on gas exchange parameters, dashed arrows were drawn between WW controls and their respective D plants for each grapevine variety, inoculation treatment (−M, +M), and environmental conditions (CATA, CETE).

In general, An (Figure 2A, Table S1) was more affected by the combination of high CO_2_ and elevated temperature (CETE) than by water deficit during the first 14 days after the imposition of drought, and this fact was more evident in T than in CS. Tempranillo had increased photosynthesis under CETE conditions. The most significant reduction in CO_2_ exchange caused by the water deficit was observed in −M T under ambient CO_2_ and temperature (CATA). Water deficit also reduced photosynthetic rates in +M CS two weeks after the imposition of the first drought cycle. In the T variety, gs was significantly influenced by the following factors: ‘ambient’ (*p* ≤ 0.001), AMF (*p* ≤ 0.001), ‘water availability’ (*p* ≤ 0.01), and the interaction between ‘ambient’ and ‘AMF’ (*p* ≤ 0.01) at an early stage (7 days after the onset of drought) (Table S1). Conversely, in CS at that moment, only ‘water availability’ had a significant effect on gs. However, gs in CS was influenced by AMF (*p* ≤ 0.001), water availability (*p* ≤ 0.001), and the interaction between ambient conditions and AMF two weeks after the onset of drought. The lowest values of leaf conductance (gs) were measured in +M T and CS two weeks after the beginning of the drought treatment under CETE conditions (Figure 2B). Under CATA conditions, the water deficit reduced gs in both −M and +M plants of both varieties. Transpiration (E) (Figure 2C) was more sensitive to drought stress under CETE than under CATA conditions, especially in CS, which is the variety whose E significantly decreased from the first week of the imposition of drought, regardless of the presence or absence of AMF. The ANOVA results (Table S1) indicate that only the factor ‘water availability’ had a significant effect on E in CS from the first week after the onset of drought (*p* ≤ 0.001). However, in both grapevine varieties, the lowest E under CETE conditions was observed in +M plants two weeks after the onset of drought. At that moment, AMF had a clear influence on E in CS (*p* ≤ 0.001), and the triple interaction between AMF, ambient, and water availability had a significant effect on E in T plants (*p* ≤ 0.001) (Table S1). Moreover, the application of the mixture of AMF and PGPR favored the maintenance of water in tissues when water deficit was imposed under CATA conditions: only +M T and CS plants reduced their E as a consequence of drought (Figure 2C).

In both grapevine varieties, the factor ‘ambient’ had a significant effect on the intercellular CO_2_ concentration (Ci) (*p* ≤ 0.001) (Table S1). Ci (Figure 2D) was, as expected, higher under CETE than under CATA conditions in both T and CS, regardless of the water regime and AMF inoculation. However, the strongest effect of drought on Ci was observed under CETE conditions: +M T plants reduced their Ci 7 days after the imposition of the first drought cycle, and −M and +M CS reduced their Ci two weeks after the onset of water deficit (Figure 2D). Under CATA conditions, Ci decreased only in +M CS as a consequence of the water deficit, with this effect being evident from the first week after the first drought cycle. The impact of the factors ‘ambient’, ‘AMF’, and ‘water availability’ and their interactions on Ci was more evident in CS than in T (Table S1).

**Figure 3:**
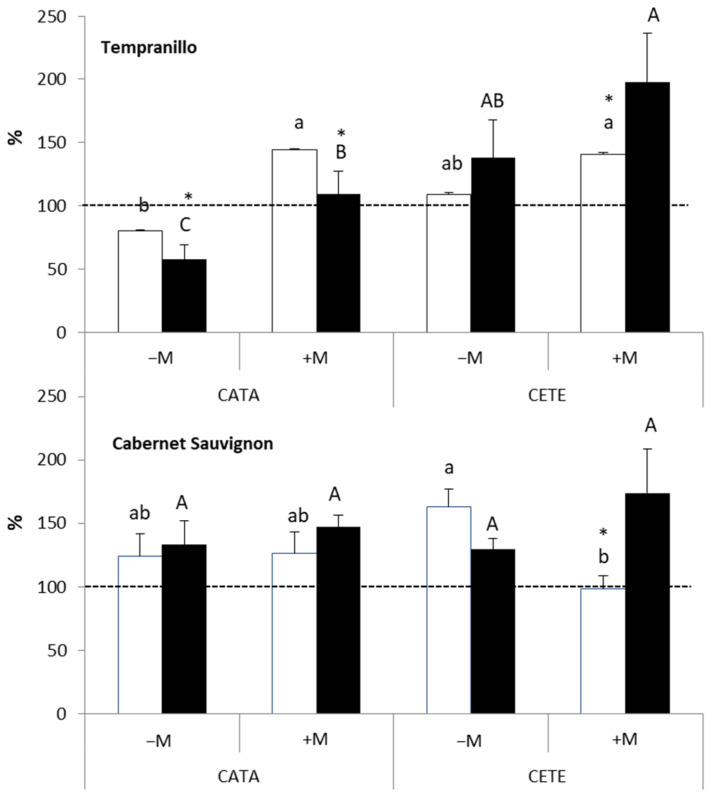
Effect of water deficit on the instantaneous water use efficiency (*WUE*) on days 7 (white bars) and 14 (black bars) after the onset of drought in Tempranillo and Cabernet Sauvignon, either inoculated (+M) or not (−M) with AMF and cultivated under current (CATA) or predicted (CETE) environmental conditions. Results are expressed as percentages of the respective WW controls (100%, dashed line). Bars represent means (*n* = 4) ± SD. For each grapevine variety, different lowercase and capital letters indicate significant differences (*p* ≤ 0.05) on days 7 or 14, respectively. Asterisks indicate significant differences (*, *p* ≤ 0.05) between days 7 and 14.

In general, *WUE* increased in plants subjected to drought (Figure 3). In T, the increase was more evident in +M plants 7 days after the imposition of water deficit under CATA conditions and 14 days after the beginning of drought under CETE conditions. The effect of the ‘ambient’ factor on the *WUE* of drought-stressed T plants was only observed 14 days after the onset of the water deficit (*p* ≤ 0.001). However, the impact of AMF on the *WUE* of water-stressed T was significant 7 days earlier (*p* ≤ 0.01) (Table S2). Similar results were observed in CS, where even −M plants increased their *WUE* when exposed to drought. However, AMF significantly affected *WUE* on days 7 (*p* ≤ 0.05) and 14 (*p* ≤ 0.01) after the onset of drought. In both varieties, the highest *WUE* value was measured 14 days after the onset of drought and corresponded to +M plants grown under CETE conditions. 

**Figure 4:**
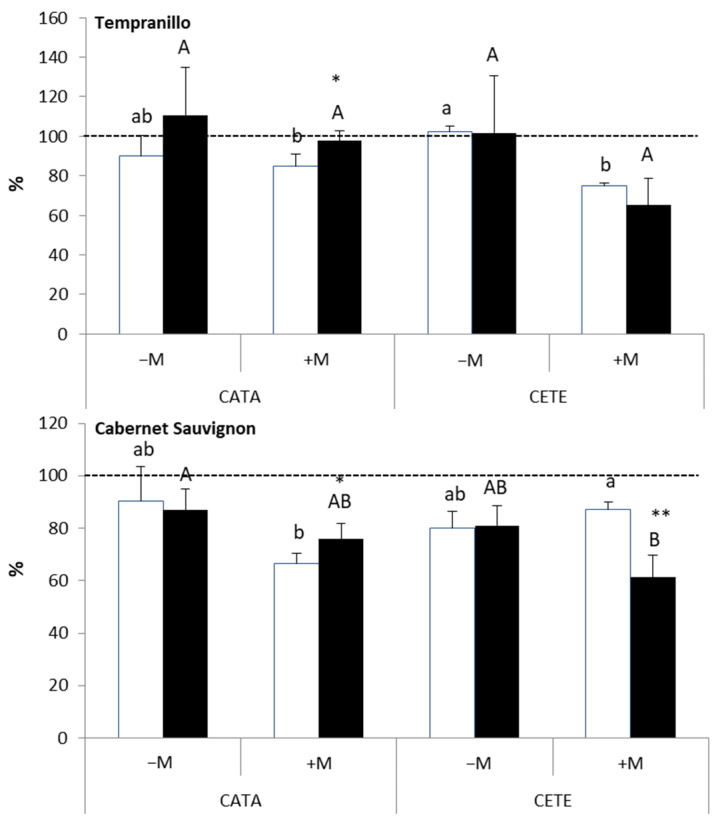
Effect of water deficit on the ratio between intercellular (Ci) and atmospheric (Ca) CO_2_ on days 7 (white bars) and 14 (black bars) after the onset of drought in Tempranillo and Cabernet Sauvignon, either inoculated (+M) or not (−M) with AMF and cultivated under current (CATA) or predicted (CETE) environmental conditions. Results are expressed as percentages of the respective WW controls (100%, dashed line). Bars represent means (*n* = 4) ± SD. For each grapevine variety, different lowercase and capital letters indicate significant differences (*p* ≤ 0.05) on days 7 or 14, respectively. Asterisks indicate significant differences (*, *p* ≤ 0.05; **, *p* ≤ 0.01) between days 7 and 14.

Figure 4 shows the ratio between the intercellular CO_2_ concentration (Ci) and atmospheric CO_2_ concentration (Ca) (results are also expressed as a percentage of WW controls). The lowest values (60–80% of WW controls) were measured in +M plants of the two grapevine varieties 14 days after the beginning of the drought cycle under CETE conditions. AMF strongly affected this ratio in drought-treated T. A significant effect was found on days 7 (*p* ≤ 0.01) and 14 (*p* ≤ 0.001) after the onset of water stress (Table S2), and there was also a significant interaction between the factors ‘AMF’ and ‘ambient’ (*p* ≤ 0.05). The effect of AMF on the Ci/Ca ratio in CS under water deficit was not clear until the second week after the imposition of drought (*p* ≤ 0.01).

**Figure 5:**
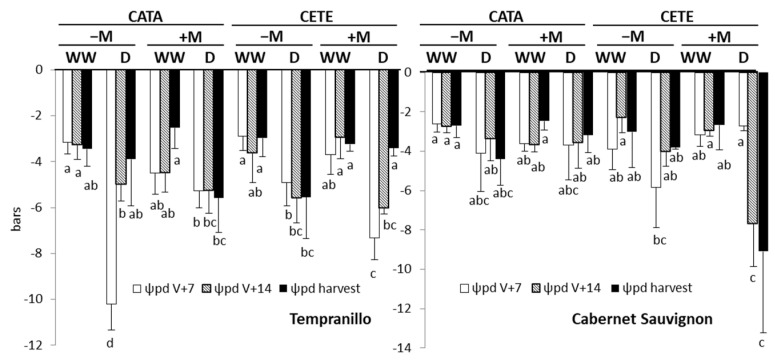
Predawn leaf water potential (Ψ_pd_) on days 7 (white bars) and 14 (grey bars) after the onset of drought and at fruit harvest (black bars) in Tempranillo and Cabernet Sauvignon, either inoculated (+M) or not (−M) with AMF, well-watered (WW) or subjected to drought (D), and cultivated under current (CATA) or predicted (CETE) environmental conditions. Bars represent means (*n* = 4) ± SD. For each grapevine variety and day, different letters indicate significant differences (*p* ≤ 0.05).

‘Water availability’ (*p* ≤ 0.001) and the interactions between ‘AMF’ and ‘ambient’ (*p* ≤ 0.001), ‘AMF’ and ‘water availability’ (*p* ≤ 0.01) and the triple interaction between ‘AMF’, ‘ambient’ and ‘water availability’ (*p* ≤ 0.001) had an early effect on Ψ_pd_ in T (Table S1). Under CATA conditions, water deficit reduced Ψ_pd_ in the leaves of T to a greater extent than in CS (Figure 5), with this reduction being particularly pronounced in −M plants 7 days after the onset of drought compared to their respective WW controls. In CS, only the interaction between ‘AMF’ and ‘water availability’ significantly affected Ψ_pd_ 7 days after the onset of drought (*p* ≤ 0.05). The factors ‘ambient’, ‘AMF’, and ‘water availability’ significantly affected Ψ_pd_ two weeks after the drought was imposed in this grapevine variety. In this variety, the greatest reduction in Ψ_pd_ was observed in +M plants 14 days after the onset of the water deficit under CETE conditions.

**Figure 6:**
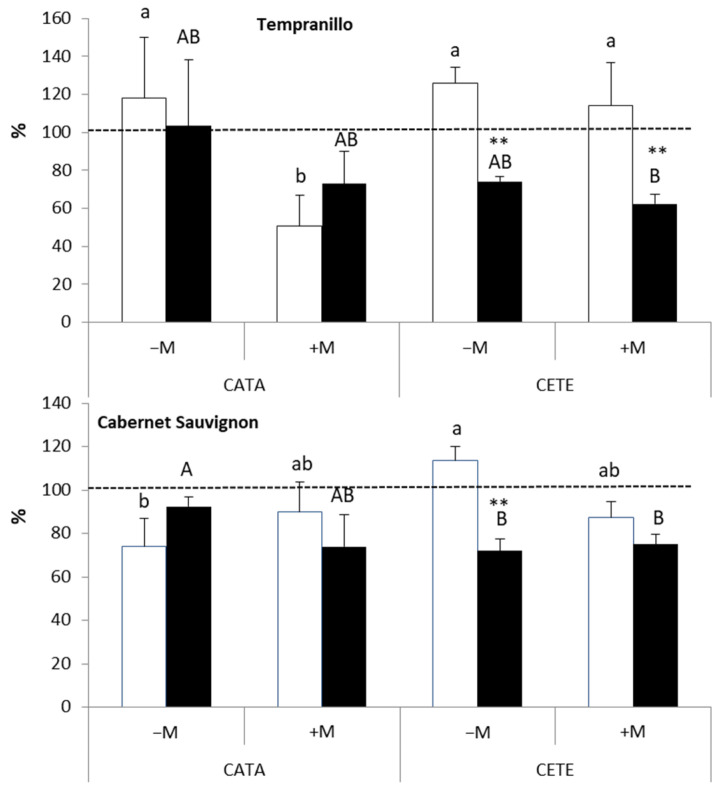
Effect of water deficit on plant hydraulic conductance (Kh) on days 7 (white bars) and 14 (black bars) after the onset of drought in Tempranillo and Cabernet Sauvignon, either inoculated (+M) or not (−M) with AMF and cultivated under current (CATA) or predicted (CETE) environmental conditions. Results are expressed as percentages of the respective WW controls (100%, dashed line). Bars represent means (*n* = 4) ± SD. For each grapevine variety, different lowercase and capital letters indicate significant differences (*p* ≤ 0.05) on days 7 or 14, respectively. Asterisks indicate significant differences**, *p* ≤ 0.01) between days 7 and 14.

Figure 6 shows the change in the plant hydraulic conductance (Kh) as a result of the water deficit on days 7 and 14 after the imposition of drought (results expressed as a percentage of the corresponding WW control). Under CATA conditions, Kh decreased 7 days after the application of the first cycle of the water deficit in +M T plants, reaching about 60% of their respective WW controls in contrast to the almost 20% increase found in −M T plants. The effect of AMF on the Kh of drought-stressed T was significant (*p* ≤ 0.01) one week after the onset of the water deficit (Table S2). In contrast, in CS, the earliest decrease in Kh was observed in −M plants (Figure 6), and AMF did not significantly affect Kh until two weeks after the onset of drought (*p* ≤ 0.05) (Table S2). Under CETE conditions, one week after the imposition of drought, Kh increased above their respective well-watered controls in −M (30%) and +M (10%) T plants, as well as in −M CS plants (10%). However, two weeks after the onset of drought, Kh decreased in both varieties (reaching values between 60 and 80% in their respective WW controls), regardless of AMF inoculation.

**Figure 7:**
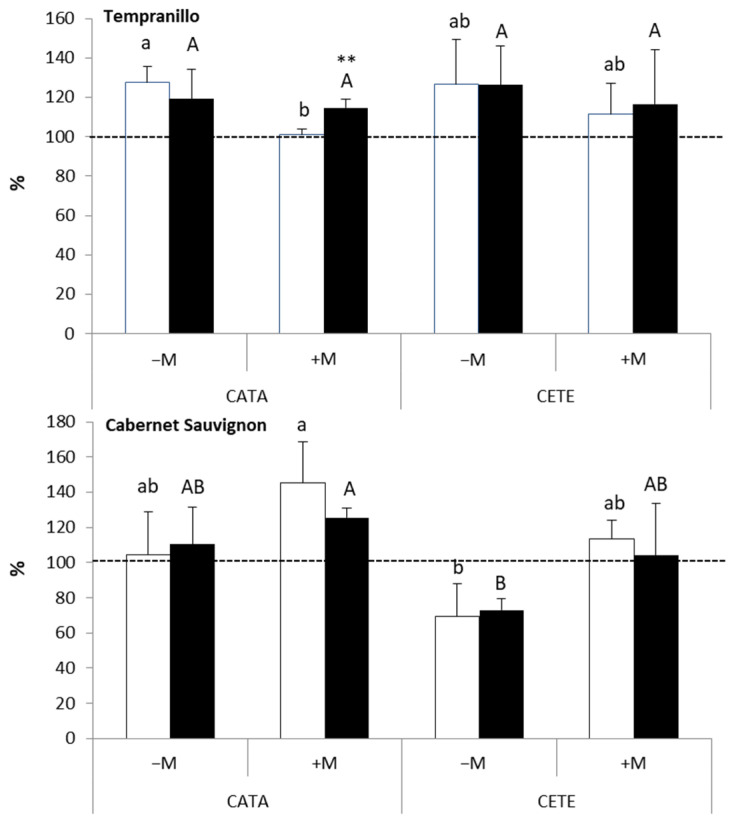
Effect of water deficit on leaf water content (WC) on days 7 (white bars) and 14 (black bars) after the onset of drought in Tempranillo and Cabernet Sauvignon either inoculated (+M) or not (−M) with AMF and cultivated under either current (CATA) or predicted (CETE) environmental conditions. Results are expressed as percentages of the respective WW controls (100%, dashed line). Bars represent means (*n* = 4) ± SD. For each grapevine variety, different lowercase and capital letters indicate significant differences (*p* ≤ 0.05) on days 7 or 14, respectively. Asterisks indicate significant differences (**, *p* ≤ 0.01;) between days 7 and 14.

In T, the amount of water (WC) in the leaf tissue did not change significantly as a result of the water deficit either in CATA or in CETE conditions (Figure 7). Only in leaves of −M CS under CETE conditions did drought reduce WC early, reaching values of about 80% of the WW controls. The ANOVA results (Table S2) indicate that CS is more sensitive to drought than T in terms of leaf WC. Both ‘AMF’ and ‘ambient’ factors significantly affected this parameter on days 7 (AMF: *p* ≤ 0.001; ambient: *p* ≤ 0.01) and 14 (AMF: *p* ≤ 0.01; ambient: *p* ≤ 0.01) after the imposition of the water deficit to CS, while it was only affected by AMF in T on day 14 (*p* ≤ 0.01).

ANOVA results (Table S1) showed that the levels of proline in leaves were significantly affected by the factors ‘ambient’ (*p* ≤ 0.001) and ‘AMF’ (*p* ≤ 0.05) and their interaction (*p* ≤ 0.001) in T one week after the onset of drought. At this point, ‘AMF’ was the most influential factor affecting the concentration of proline in the leaves of CS (*p* ≤ 0.01), along with its interactions with ambient (*p* ≤ 0.001) and water availability (*p* ≤ 0.001). One week later, the factor ‘AMF’ had an impact on the levels of proline in both grapevine varieties, either alone or in combination with water availability. Two weeks after drought imposition, proline levels decreased in +M T under CATA conditions and in −M T under CETE conditions compared to their respective WW controls (Table 1). In contrast, proline accumulated in leaves of +M CS 7 days after the onset of the water deficit under CATA conditions. Under CETE conditions, −M CS had a lower amount of proline than +M CS, regardless of the water regime. 

The application of different factors did not have a significant effect on the levels of TSS in CS 7 days after the drought was imposed. At this point, the factor ‘AMF’ had a significant effect on the accumulation of TSS in the leaves of T (*p* ≤ 0.001), similar to its interactions with ‘ambient’ (*p* ≤ 0.001) and ‘water availability’ (*p* ≤ 0.05) (Table S1). Seven days after the imposition of drought, the concentration of TSS significantly increased in the leaves of +M T under CATA conditions compared to their respective WW controls (Table 1). One week later (14 days after the onset of drought), only ‘ambient’ conditions and the interaction between ‘ambient’ conditions and ‘water availability’ affected the concentration of TSS in leaves of T and CS, respectively (Table S1).

#### 2.2.2. Late Responses

In T, the strongest effect of water deficit on leaf Ψ_pd_ at fruit maturity was found in +M plants under CATA conditions and in −M plants under CETE conditions. Ψ_pd_ in the leaves of these plants reached values approximately 3 and 2 bars lower than those measured in their respective WW controls (Figure 5). In CS, the greatest reduction in Ψ_pd_ was observed in +M plants under CETE conditions.

**Figure 8:**
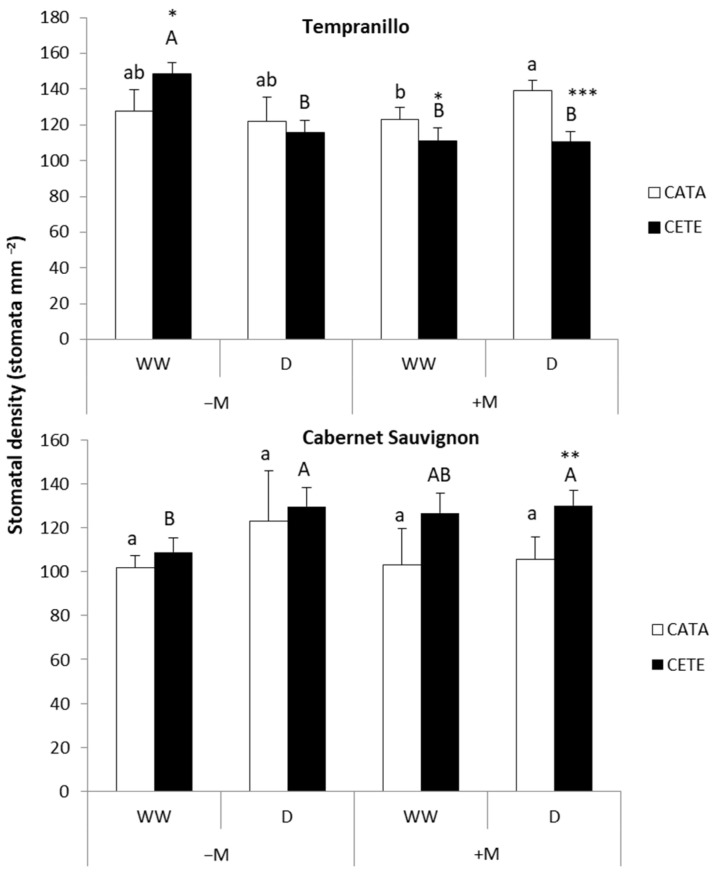
Stomatal density in leaves of Tempranillo and Cabernet Sauvignon, either inoculated (+M) or not (−M) with AMF, well-watered (WW) or subjected to drought cycles (D) and cultivated under current (CATA, white bars) or predicted (CETE, black bars) CO_2_ concentrations and air temperatures. Data were collected at the fruit harvest. Values are means (*n* = 4) ± SD. For each grapevine variety, different lowercase and capital letters indicate significant differences (*p* ≤ 0.05) under CATA or CETE conditions, respectively. Asterisks indicate significant differences (*, *p* ≤ 0.05; **, *p* ≤ 0.01; ***, *p* ≤ 0.001) between CATA and CETE.

The most interesting results concerning the effect of drought on stomatal density (SD) were found in +M plants (Figure 8). In these plants, the presence of AMF (+M) induced changes in SD when comparing CATA and CETE conditions, with different results in the two grapevine varieties. While the water deficit decreased SD in T under CETE conditions, it increased SD in CS. The measurements of stomatal length, width, and area revealed that the size of the stomata in +M CS exposed to drought was smaller under CETE than under CATA conditions (Table 2). In contrast, the size of the stomata in +M T subjected to the water deficit was similar under these two environmental conditions.

## 3. Discussion

### 3.1. Early Responses to Drought

Under CATA conditions, the Kh of +M T plants decreased 7 days after the imposition of drought compared to its well-watered control, which can be explained by a significant decrease in E and the maintenance of Ψ_pd_. However, in −M T plants, E did not decrease significantly, in contrast to the strong decrease in Ψ_pd_, which favored the maintenance of Kh at values similar to the well-watered control. This decrease in Ψ_pd_ was not associated with an active osmotic adjustment since no accumulation of proline and/or total soluble sugars was detected. All these results suggest different physiological mechanisms in the −M and +M of Tempranillo plants in the early stages of water deficit. The marked decrease in Ψ_pd_ in −M plants could be the result of a passive osmotic adjustment caused by the water efflux from the symplast to the apoplast [19]. This passive osmotic adjustment would have allowed them to concentrate the present solutes in a smaller volume of water, this being an appropriate strategy given that the photosynthetic rates of these plants decreased rapidly in a drought situation. In +M plants, the maintenance of Ψ_pd_ suggests an adjustment of the elasticity of the cell wall [19], something already observed by Goicoechea et al. [20] in alfalfa plants, where the mycorrhizal association induced a decrease in the elastic modulus under restricted irrigation conditions. However, under conditions of high CO_2_ and elevated temperature (CETE), the behavior of +M plants in the early stages of drought was different. Although E was reduced, a marked decrease in Ψ_pd_ was observed, leading to high Kh values. This decrease in Ψ_pd_ could be due to the higher concentration of total soluble sugars detected in the leaves of +M plants under drought, which is favored by the maintenance of high photosynthetic rates, indicating greater stress tolerance in +M plants [21]. The accumulation of solutes is quite common in grapevines under water stress [22]. However, the high Kh values were not maintained over time since a significant drop in Kh was observed one week later in +M T plants, associated with a marked decrease in leaf gs and E. This behavior at slightly late stages could be due to changes in the expression of certain genes, including those encoding aquaporins and abscisic acid (ABA). Porcel et al. [15] observed changes in the expression of the *PIP* aquaporin gene in lettuce and soybeans exposed to ten days of drought. Although our results contradict the idea that AMF can increase soi–-plant hydraulic conductance under drought [14], Bárzana et al. [23] concluded that AMF can either up- or down-regulate genes related to aquaporin expression in the roots of their host plants depending on the water conditions and the severity and duration of water deficit periods, thus modulating the root’s hydraulic conductance and the plant’s water status. Furthermore, Cochetel et al. [21] reported that grapevine varieties with higher tolerance to drought exhibit a more rapid response of the transcriptome to the water deficit and an increase in ABA biosynthesis. A higher hydraulic resistance may confer an adaptive advantage to grapevine during the water deficit by preventing rapid soil water depletion [12]. Hochberg et al. [24] also observed a down-regulation of leaf hydraulic conductance in grapevines during water deficit acclimation. They hypothesized that this down-regulation was not related to embolism but rather to leaf turgor and membrane permeability. Albuquerque et al. [25] attributed the reduced leaf hydraulic and stomatal conductance of grapevine under a moderate water deficit to changes in the outside-xylem pathways. Specifically, they found a decrease in membrane permeability associated with a Casparian-like band in the leaf vein bundle sheath rather than xylem embolism.

Although grafted on the same rootstock, the CS variety showed a different behavior, especially under CATA conditions: only small changes (in −M plants) or no significant changes (in +M plants) in Kh were observed 7 days after the application of drought compared to their WW controls. This was likely due to the fact that Ψ_pd_ hardly changed as a result of water stress. In +M plants, however, a decrease in E was observed, which was associated with stomatal closure. Furthermore, only in +M plants was there a tendency for E to continue to decrease during the second week of the water deficit. This suggests that beneficial rhizospheric microorganisms (AMF + PGPR) provided CS with the ability to respond more rapidly to drought, which may have increased its adaptive capacity. Under CETE conditions, the behavior of CS was similar to that of T, as Kh decreased in −M and +M plants after 14 days of drought, which could be attributed to the decrease in E. However, +M plants also showed a strong decrease in Ψ_pd_, which occurred after 14 days of drought. Although proline levels were higher in +M than in −M plants, no active osmotic adjustment was observed in +M CS as a consequence of drought. This suggests that the decrease in Ψ_pd_ observed in +M plants was likely due to changes in the distribution of water between the apoplast and symplast, as previously observed in mycorrhizal alfalfa, whose percentage of apoplastic water increased under the water deficit [20]. This presumed that the passive concentration of solutes in the symplast would have coincided with reduced photosynthetic rates in +M CS. The plasticity exhibited by +M plants in the face of drought under CETE conditions allowed them to more efficiently maintain a leaf water content, which was similar to that of their respective WW controls in contrast to the 20–30% decrease in leaf water content observed in −M plants. The drought tolerance of CS is not very high [26], but acclimation improves the resistance of this variety to recurrent water deficits [27]. Lehr et al. [28] concluded that the proline concentration in Cabernet Sauvignon leaves and the expression of *P5CS*, the gene encoding the enzyme that catalyzes the first two steps of proline biosynthesis, are good markers of combined drought and heat stress in this grape variety. However, our results suggest that the concentration of proline may be a good marker of drought in +M plants and that elevated CO_2_ may nullify the effect of combined drought and heat on proline accumulation. The increased resilience to drought observed in +M CS may be mediated by strigolactones [29]. Plants can enhance the biosynthesis of these signaling molecules to favor the establishment of mycorrhizal symbiosis to withstand water deficits [30]. Therefore, the association of CS with AMF could enhance plant adaptability and resilience during the acclimation process to water deficits under climate change.

Under CETE conditions, mycorrhizal symbiosis (+M) also improved the *WUE* of T and CS when exposed to drought. This benefit was evident after two weeks of drought and was accompanied by a lower Ci to Ca ratio. Kelly et al. [31] proposed the ‘low Ci effect’ as a mechanism by which elevated CO_2_ in the air can mitigate the effects of drought on woody plants: stomata closure under water deficit reduces the intercellular CO_2_ concentration (Ci), resulting in a greater relative enhancement of the photosynthesis under elevated atmospheric CO_2_.

### 3.2. Late Responses to Drought

The stomatal density (SD) in the leaves of CS was higher than that reported by Boso et al. [32], while the size of the stomata (SS) was smaller than that measured by these authors. These differences may be due to the fact that Boso et al. [32] carried out the measurements on adult vines in the field, while in our study, stomatal characteristics were studied on young leaves of 3-year-old plants. As mentioned in the results section, the most striking data in our study were the change induced by drought in SD in the leaves of T and CS associated with AMF (+M) when grown under CETE conditions compared to the values observed under CATA conditions. Given that the stomatal parameters were measured at the time of fruit maturity, that is, approximately two and a half months after the beginning of the drought, it is reasonable to think that mature leaves perceived the signs of water scarcity, high temperature, and high CO_2_. This would have induced a systemic response on the development of stomata in the epidermis of the leaves developed during that period [33]. However, the response to T and CS was opposite as follows: in +M T, water deficit was linked to elevated temperature and high CO_2_ reduced SD, which is frequently observed under high CO_2_ levels [27], but SD was increased in +M CS. In the T variety, drought did not change the SS under CETE conditions. However, SS significantly decreased in CS under the same environmental conditions. Driesen et al. [33] also observed increased SD and reduced SS (decreased length) in basil leaves under a water deficit. Anatomical adjustments related to stomatal parameters can influence gas exchange [33] and *WUE* [34] without sacrificing biomass production [33]. Reducing SD is considered a conservative strategy to prevent significant water loss through transpiration, which usually results in greater *WUE*. Studies on *Arabidopsis* and poplar have shown that the overexpression of *AtEPF2* and *PdEPFL6* genes, respectively, leads to a reduction in SD [34,35]. On the other hand, a smaller SS may confer a greater ability to open and close the stomata more quickly [36]. To the best of our knowledge, this is the first time that a study suggests an effect of mycorrhization on the expression of genes related to the anatomical plasticity of stomatal parameters in response to medium-term drought. Subsequent studies aim to confirm this hypothesis.

## 4. Materials and Methods

### 4.1. Plant Material and Growth Conditions

One-year-old Tempranillo (T) and Cabernet Sauvignon (CS) plants, grafted onto R110 rootstock, were grown in 13-L pots filled with a mixture of peat, vermiculite, and sand (1:2.5:2.5). The peat used (Floragard, Vilassar de Mar, Barcelona) contained nitrogen (70–150 mg L^−1^), P_2_O_5_ (80–180 mg L^−1^) and K_2_O (140–220 mg L^−1^), had a pH of 5.2–6.0 and was previously sterilized at 100 °C for 1 h on three consecutive days. The plants were divided into the following two groups: (1) half of the plants of each grapevine variety received 10 g (per plant) of a commercial inoculum (Bioradis Plant, Bioera SLU, Tarragona, Spain) containing approximately 100 spores per gram of a mixture of five AMFs (*Rhizophagus irregularis*, *Funneliformis mosseae*, *Septoglomus deserticola*, *Claroideoglomus claroideum* and *Claroideoglomus etunicatum*) accompanied by 4 × 10^7^ of CFU (Bacteria Forming Unit) per gram of a mixture of four PGPRs (*Bacillus subtilis, B. megaterium, B. altitudinis* and *B. licheniformis*) (+M plants); (2) the other half of the plants of each grapevine variety only received a filtrate containing the rhizobacteria (−M plants). The filtrate was obtained by washing an equivalent amount of the inoculum with distilled water and vacuum-filtering the resulting liquid through 15–20 mm diameter filters with a particle retention capacity of 2.5 µm (Whatman 42; GE Healthcare, Little Chalfont, UK). With this, the differences between −M and +M plants could be attributed exclusively to the absence or presence of AMF. The plants were inoculated following the same protocol in the second and third years.

When plants were three years old (in 2023), 32 plants from each grapevine variety were grown in temperature gradient greenhouses (TGGs) under two environmental conditions applied from fruit set (E-L 27) to maturity (E-L 38) [37]: (1) current CO_2_ and temperature conditions (CATA, ca. 400 ppm CO_2_ and ambient air temperature corresponding to the summer of 2023 (Table 3) (8 −M and 8 +M plants of each variety) or (2) climate change conditions predicted by the year 2100 (CETE, 700 ppm CO_2_ and ambient air temperature +4 °C) (8 −M and 8 +M plants of each variety). The increase in temperature to 4 °C with respect to the current ambient values implemented in the CETE treatment, was chosen in order to simulate the changes projected for the end of the 21st century, as per the SSP5-8.5 greenhouse emissions scenario derived from the concentration-driven CMIP6 model simulations [38]. At fruit veraison (E-L 35), within each environmental condition (CATA or CETE), half of the plants of every variety (T or CS) and inoculation treatment (−M or +M) were divided into two homogeneous groups and subjected to two levels of water availability: maintained full irrigation (WW) (90–100% substrate field capacity, FC) or restricted irrigation (D) (cycles from 90–100% till 20–30% FC). The soil’s water content was monitored using EC 5 water sensors (Decagon Devices, Inc., Pullman, WA, USA). Therefore, the total number of treatments applied to each grapevine variety was eight (−M/+M plants, CATA/CETE conditions, and WW/D water regime: 2 × 2 × 2). The number of biological replicates (plants) per variety and treatment was four. Plants were regularly pruned and irrigated with alternating water and nutrient solution [39].

### 4.2. Physiological and Biochemical Determinations

Both pre-dawn (Ψ_pd_) and midday (Ψ_md_) leaf water potentials were measured in young fully expanded leaves (four leaves for each variety and treatment) on days 7 and 14 after veraison (therefore, 7 and 14 days after the imposition of the first drought cycle). A SKYE SKPM 1400 pressure chamber (Skye Instruments Ltd., Llandrindod, Wales) was used. Ψ_pd_ was also measured at fruit maturity. Photosynthesis (An), leaf conductance (gs), transpiration rates (E), and intercellular CO_2_ concentrations (Ci) were measured inside the TGGs, 7 and 14 days after veraison with a portable photosynthesis system (ADC-LCi, BioScientific Ltd., Hoddesdon, UK). The measurements were taken in fully developed young leaves from 10.00 to 12.00 h with a photosynthetically active photon flux density (PPFD) of 1200 µmol m^−2^ s^−1^. Gas exchange measurements were performed at the CO_2_ concentration, temperature, and relative humidity corresponding to the growing conditions of each plant. Whole plant hydraulic conductance (Kh) was estimated as Kh = E/(Ψ_pd_ − Ψ_md_), considering Kh from Ohm’s law analogy for the soil–plant–atmosphere continuum [41], where E, Ψ_leaf_, and Ψ_soil_ are the transpiration rate, leaf water potential and soil water potential, respectively. Ψ_pd_ was taken as a proxy for Ψ_soil_, and Ψ_md_ was taken as Ψ_leaf_. Instantaneous water use efficiency (*WUE*) was calculated as the ratio between An and E. The ratios of intercellular (Ci) to atmospheric (Ca) CO_2_ were also calculated [31]. Proline and total soluble sugars (TSSs) were measured, as described by Goicoechea et al. [42], in the same leaves previously used to determine Ψ_pd_. Leaf dry weight (DW) was calculated after drying the plant material in an oven at 70 °C until reaching a constant weight. Leaf water content (WC) was calculated as (leaf fresh weight−leaf dry weight)/leaf dry weight.

### 4.3. Stomatal Anatomy

At fruit maturity (approximately 2.5 months after the onset of the water deficit), dental resin impressions were used to study the anatomical characteristics of the stomata located at the abaxial leaf surfaces. A fresh resin was applied at the point of the maximum leaf width near the central vein. The dental resin mold was covered with nail polish to create a cast that was examined under a light microscope (Olympus CX40, Olympus Iberia S.A.U., L’Hospitalet de Llobregat, Barcelona, Spain). Four to five leaves and five microscopic fields of each epidermal surface impression were randomly examined from each treatment. Stomatal density (SD) was determined in plants from all eight treatments applied in the study. Stomatal size (SS) was only measured in +M plants from each grapevine variety grown under either CATA or CETE conditions and subjected to drought (D). SS included stomatal length, stomatal width, and stomatal area. Stomatal length and width were measured using a microscope with a 40× objective magnification. The stomatal area was estimated by attributing an ellipse shape to each stoma.

### 4.4. Statistical Analysis

Data were analyzed using the XLSTAT 7.5.2 statistical software. The data on photosynthesis, leaf conductance, transpiration, intercellular CO_2_ concentration, pre-dawn leaf water potential, and concentrations of proline and total soluble sugars were analyzed using a three-way ANOVA. The main factors were ‘ambient (CATA or CETE, amb)’, ‘arbuscular mycorrhizal fungi, AMF’, and ‘water availability, water’. The study also applied a two-way ANOVA to determine the effects of the main factors ‘arbuscular mycorrhizal fungi, AMF’ and ‘ambient, CATA or CETE, amb’ and their interaction on the values, expressed as percentages, of the well-watered controls of instantaneous water use efficiency, the intercellular to ambient CO_2_ ratio, plant hydraulic conductance, and leaf water content in drought-stressed plants. Student’s *t*-test was applied to each of the two samples, assuming unequal variances. The degree of significance was set at *p* ≤ 0.05. The Bonferroni correction (0.05/*n*) was then applied, being *n* = the total number of comparisons made with Student’s *t*-test.

## 5. Conclusions

This study reports the early and late physiological and anatomical responses of two grapevine varieties subjected to drought during fruit ripening. Although both Tempranillo (T) and Cabernet Sauvignon (CS) were grafted onto the same type of rootstock, some responses were variety-dependent. In the early stages following the onset of the water deficit, T exhibited greater sensitivity than CS to environmental conditions such as CO_2_ concentration, temperature, and water availability, as well as to mycorrhizal symbiosis. These abiotic factors significantly impacted the gas exchange parameters, including photosynthesis, leaf conductance, transpiration, and intercellular CO_2_ concentration, while mycorrhization affected *WUE*, the Ci/Ca ratio, and plant Kh in drought-stressed plants. During the early stages, water availability primarily determined leaf conductance, transpiration, and intercellular CO_2_ concentration in the case of CS. However, mycorrhization became increasingly important for CS as it significantly affected gas exchange parameters, pre-dawn water potential, and proline accumulation in the leaves. Additionally, it impacted *WUE*, the Ci/Ca ratio, plant Kh, and leaf WC in drought-stressed plants. In T, the greatest influence of mycorrhization on the gas exchange parameters and proline accumulation after two weeks of drought was observed in the interaction with abiotic factors, such as environmental conditions and soil water availability. Moreover, in both grapevine varieties, the mycorrhizal association induced changes in leaf anatomy that became apparent just over two months after drought establishment. These changes were observed in leaves that developed under climate change conditions, including high CO_2_, high temperature, and drought. The plants adapted to reduced water loss by transpiration. In T, the change resulted in a decrease in stomatal density, whereas in CS, the change led to a decrease in stomatal size, which suggests that mycorrhizae may regulate the expression of genes related to leaf anatomy.

Despite the different responses of each of the grapevine varieties studied, we can confirm that the interaction of the rootstock with AMF was a key factor for the grapevine’s adaptation to the water deficit under both current and future environmental conditions.

## Figures and Tables

**Table 1:** Concentrations of proline (µmol g^−1^ DW) and total soluble sugars (TSSs) (mg g^−1^ DW) in leaves of Tempranillo (T) and Cabernet Sauvignon (CS), either inoculated (+M) or not (−M) with AMF, well-watered (WW) or subjected to drought (D) and cultivated under current (CATA) or predicted (CETE) CO_2_ concentrations and air temperature. Data were collected on days 7 and 14 after the onset of the water deficit. Values are means (*n* = 4). For each organic solute (proline or TSS), grapevine variety (T or CS), environmental conditions (CATA or CETE), and day (7 or 14), different letters indicate significant differences (*p* ≤ 0.05). DW = dry weight.

				Proline		TSS	
				7 Days	14 Days	7 Days	14 Days
T	CATA	−M	WW	2.49 a	1.29 b	68.17 b	89.78 a
			D	1.77 a	1.29 b	53.31 b	108.00 a
		+M	WW	1.29 a	2.28 a	104.87 ab	112.75 a
			D	1.37 a	0.96 b	118.65 a	104.61 a
	CETE	−M	WW	0.48 AB	1.33 A	85.38 AB	114.11 A
			D	0.26 B	0.30 B	98.52 AB	129.83 A
		+M	WW	0.49 AB	1.57 A	84.18 B	109.19 A
			D	0.56 A	0.81 A	101.27 A	118.34 A
CS	CATA	−M	WW	0.43 b	1.01 a	100.75 a	115.47 a
			D	0.35 b	1.07 a	108.65 a	132.07 a
		+M	WW	0.52 b	1.72 a	98.38 a	125.23 a
			D	1.13 a	1.30 a	113.15 a	127.65 a
	CETE	−M	WW	0.90 A	0.52 B	105.34 A	118.02 A
			D	0.31 A	0.74 B	92.32 A	106.18 A
		+M	WW	0.55 A	1.40 A	101.76 A	134.57 A
			D	0.59 A	1.13 A	113.16 A	101.17 A

**Table 2:** Stomatal length, width, and area in +M Tempranillo (T) and +M Cabernet Sauvignon (CS) subjected to drought under CATA or CETE conditions. Data were obtained at fruit harvest (around 2.5 months after the onset of drought cycles). Values are means (*n* = 20–25 stomata from 4 plants per treatment). For each parameter, different letters indicate significant differences (*p* ≤ 0.05).

	Stomatal Length (µm)		Stomatal Width (µm)		Stomatal Area (µm^2^)	
	CATA	CETE	CATA	CETE	CATA	CETE
T	28.00 a	28.88 a	18.25 a	17.75 a	404.08 a	406.97 a
CS	28.75 a	26.60 a	19.44 a	15.20 b	445.73 a	319.45 b

**Table 3:** Temperatures (°C) recorded in Pamplona in summer 2023 [40].

	Average of the Maximum	Average of the Minimum	Absolute Maximum	Absolute Minimum
June	26.6	15.7	33.4	12.6
July	28.8	16.3	36.3	13.5
August	30.7	15.5	40.4	9.4
September	26.7	14.9	33.3	8.3

## Data Availability

The data used to support the findings of this study are available from the author upon request.

## Supplementary Materials

The following supporting information can be downloaded at: https://www.mdpi.com/article/10.3390/plants13081155/s1, Table S1: Results of the three-way ANOVA applied to leaf photosynthesis (An), stomatal conductance (gs) and transpiration (E), intercellular CO_2_ (Ci), pre-dawn leaf water potential (ψpd), and concentration of proline and total soluble sugars (TSS) in Tempranillo and Cabernet Sauvignon subjected to drought on days 7 and 14 after the onset of water stress; Table S2: Results of the two-way ANOVA applied to instantaneous water use efficiency (*WUE*), ratio between intercellular (Ci) and ambient (Ca) CO_2_, plant hydraulic conductance (Kh) and leaf water content (WC) expressed as percentages of well-watered controls in Tempranillo and Cabernet Sauvignon subjected to drought on days 7 and 14 after the onset of water stress.
