# Supplementary material for: Arbuscular Mycorrhizal Fungi Improve the Performance of Tempranillo and Cabernet Sauvignon Facing Water Deficit under Current and Future Climatic Conditions
Source: Plants (Basel). 2024 Apr 22;13(8):1155. doi: 10.3390/plants13081155 (PMC11054116; doi:10.3390/plants13081155)
Supplement: Supplementary file 1 [file plants-13-01155-s001.zip › plants-2947383-supplementary.pdf]

Table S1. Results of the three-way ANOVA applied to leaf photosynthesis (An), stomatal conductance (gs) and transpiration (E), intercellular CO_2 (Ci), pre-dawn leaf water potential (ψ_{pd}), and concentration of proline and total soluble sugars (TSS) in Tempranillo and Cabernet Sauvignon subjected to drought on days 7 and 14 after the onset of water stress. The three main factors were 'ambient (CATA or CETE, amb)', 'arbuscular mycorrhizal fungi, AMF' and 'water availability, water'. Table is showing the probability (*p*) values of each main factor and their interactions. Significant values are highlighted in bold.

	Day 7 after the onset of drought							Day 14 after the onset of drought						
	An	gs	E	Ci	ψ_{pd}	Proline	AST	An	gs	E	Ci	ψ_{pd}	Proline	AST
**Tempranillo**														
Ambient (amb)	**< 0.0001**	**< 0.0001**	**0.000**	**< 0.0001**	0.127	**< 0.0001**	0.193	**< 0.0001**	0.887	**< 0.0001**	**< 0.0001**	0.529	**0.000**	**0.036**
AMF	0.400	**0.000**	0.143	**0.003**	0.568	**0.020**	**< 0.0001**	0.120	**0.014**	0.686	**0.017**	0.347	**0.001**	0.902
Water availability (water)	**0.024**	**0.003**	**0.002**	**0.000**	**< 0.0001**	0.141	0.076	**< 0.0001**	**< 0.0001**	**< 0.0001**	0.067	**< 0.0001**	**< 0.0001**	0.181
Amb x AMF	0.119	**0.003**	0.289	0.052	**0.000**	**0.001**	**< 0.0001**	0.887	0.173	0.736	0.517	0.414	0.822	0.169
Amb x water	**0.003**	0.622	0.058	0.067	0.662	0.339	0.059	0.879	0.977	**0.032**	0.091	0.059	0.244	0.565
AMF x water	0.957	0.370	0.303	0.365	**0.007**	**0.046**	**0.050**	0.090	**0.004**	**0.003**	**0.009**	0.694	**0.012**	0.206
Ambient x AMF x water	**0.000**	0.328	0.127	0.632	**0.000**	0.334	0.187	**0.000**	**0.003**	**0.023**	0.146	0.116	**0.000**	0.443
**Cabernet Sauvignon**														
Ambient (amb)	**< 0.0001**	0.911	0.278	**< 0.0001**	0.608	0.776	0.703	**< 0.0001**	0.835	0.421	**< 0.0001**	**0.040**	**0.001**	0.182
AMF	**0.002**	0.047	0.491	**0.002**	0.141	**0.003**	0.378	**0.001**	**< 0.0001**	**< 0.0001**	**< 0.0001**	**0.020**	**< 0.0001**	0.600
Water availability (water)	0.097	**< 0.0001**	**< 0.0001**	**< 0.0001**	0.167	0.956	0.339	**0.000**	**< 0.0001**	**< 0.0001**	**< 0.0001**	**0.002**	0.243	0.322
Amb x AMF	0.188	0.088	0.584	**0.001**	0.130	**0.001**	0.490	**0.001**	**0.024**	0.666	**0.000**	0.285	0.364	0.883
Amb x water	0.216	0.947	0.427	0.908	0.615	**0.000**	0.272	**0.009**	0.185	0.161	**0.000**	**0.003**	0.355	**0.026**
AMF x water	0.746	0.057	0.149	0.295	**0.021**	**< 0.0001**	0.160	0.496	0.190	0.821	**0.018**	0.339	**0.011**	0.240
Ambient x AMF x water	0.274	0.104	0.269	**0.008**	0.815	0.816	0.424	0.458	0.251	0.676	0.186	0.203	0.976	0.850

Table S2. Results of the two-way ANOVA applied to instantaneous water use efficiency (*WUE*), ratio between intercellular (Ci) and ambient (Ca) CO_2, plant hydraulic conductance (Kh) and leaf water content (WC) expressed as percentages of well-watered controls in Tempranillo and Cabernet Sauvignon subjected to drought on days 7 and 14 after the onset of water stress. The two main factors were 'arbuscular mycorrhizal fungi, AMF' and 'ambient (CATA or CETE, amb)'. Table is showing the probability (*p*) values of each main factor and their interaction. Significant values are highlighted in bold.

	Day 7 after the onset of drought				Day 14 after the onset of drought			
	WUE	Ci/Ca	Kh	Leaf WC	*WUE*	Ci/Ca	Kh	Leaf WC
**Tempranillo**								
AMF	**0.0016**	**0.0013**	**0.0079**	0.0843	**0.0002**	**< 0.0001**	0.0826	**0.0079**
Ambient (amb)	0.4115	0.6891	0.1169	0.4169	**0.0001**	**0.0120**	0.6197	0.1169
AMF × amb	**0.0441**	**0.0411**	0.2664	0.8213	**0.0300**	**0.0554**	0.946	0.2664
**Cabernet Sauvignon**								
AMF	**0.0277**	0.0967	0.3813	**< 0.0001**	**0.0129**	**0.0028**	**0.0264**	**0.0043**
Ambient (amb)	**0.0201**	0.7081	**0.0314**	**0.0025**	0.1829	0.0527	0.1544	**0.0099**
AMF × amb	**0.0040**	**0.0436**	**0.0293**	0.6207	0.5646	0.5386	**0.0298**	0.6321
